# Reciprocal regulation between alternative splicing and the DNA damage response

**DOI:** 10.1590/1678-4685-GMB-2019-0111

**Published:** 2020-03-27

**Authors:** Adrian E. Cambindo Botto, Juan C. Muñoz, Luciana E. Giono, Nicolás Nieto-Moreno, Carmen Cuenca, Alberto R. Kornblihtt, Manuel J. Muñoz

**Affiliations:** 1 Universidad de Buenos Aires, Facultad de Ciencias Exactas y Naturales, Departamento de Fisiologia, Biologia Molecular y Celular, Instituto de Fisiologia, Biologia Molecular y Neurociencias (IFIBYNE-UBA-CONICET), Buenos Aires, Argentina.; 2 Fondazione Istituto FIRC di Oncologia Molecolare (IFOM), Milan, Italy.; 3 Universidad de Buenos Aires, Facultad de Ciencias Exactas y Naturales, Departamento de Biodiversidad y Biología Experimental, Buenos Aires, Argentina.

**Keywords:** DNA damage, alternative splicing, ATR, ATM, DNA-PK

## Abstract

Splicing, the process that catalyzes intron removal and flanking exon ligation, can occur in different ways (alternative splicing) in immature RNAs transcribed from a single gene. In order to adapt to a particular context, cells modulate not only the quantity but also the quality (alternative isoforms) of their transcriptome. Since 95% of the human coding genome is subjected to alternative splicing regulation, it is expected that many cellular pathways are modulated by alternative splicing, as is the case for the DNA damage response. Moreover, recent evidence demonstrates that upon a genotoxic insult, classical DNA damage response kinases such as ATM, ATR and DNA-PK orchestrate the gene expression response therefore modulating alternative splicing which, in a reciprocal way, shapes the response to a damaging agent.

## Introduction

A human gene is a DNA sequence that codes for a molecule with a certain function. The diversity of these molecules, including RNAs or proteins, outnumbers human genes, thereby demonstrating that amplification mechanisms of DNA information necessarily take place. The vast majority of human genes have exonic sequences (Lander *et al.*, 2001; Venter *et al.*, 2001) that are normally included in the mature RNA molecule and introns, sequences that are present in the immature RNA, or pre-RNA, but are absent in the mature RNA. Splicing, the process that catalyzes intron removal and flanking exons ligation, can occur in different ways (alternative splicing) in pre-RNAs transcribed from the same gene, thereby increasing the number of possible mature RNAs that can be obtained from a single gene. Alternative splicing (AS) is the most common mechanism that amplifies DNA encoded information since it occurs in more than the 95% of the human genes (Barash *et al.*, 2010). Genes involved in the DNA damage response (DDR) are not the exception, and many genes associated to cell cycle control, DNA repair, or controlled cell death are regulated by AS (Giono *et al.*, 2016). Regulation of gene expression by AS can modify the balance between, for instance, pro- and anti-apoptotic factors. As an example, the exclusion of an alternative sequence in the Bcl-x mRNA results in a shorter protein that acts as a dominant negative, competing with the full-length protein that has a role in promoting apoptosis (Muñoz *et al.*, 2009). Nevertheless, there exists a reciprocal regulation between AS and the DDR, since it has been recently demonstrated that classical factors involved in the DDR such as ATM (ataxia telangiectasia mutated), ATR (ataxia telangiectasia mutated and Rad3 related), and DNA-PK have a paramount role in gene expression and AS regulation in a genotoxic scenario.

### Alternative splicing regulation

Splicing, and therefore AS, is mainly a co-transcriptional process (Kotovic *et al.*, 2003; Lacadie and Rosbash, 2005; Listerman *et al.*, 2006; Pandya-Jones and Black, 2009; Ameur *et al.*, 2011; Tilgner *et al.*, 2012) and, as such, is regulated not only by factors that bind to the pre-RNA but also by the transcription process itself (Kornblihtt *et al.*, 2013). In other words, transcription and splicing are coupled processes that therefore can regulate each other. Nowadays there is a big corpus of data on the functional coupling between transcription and splicing, but one of the most clear pieces of evidence still comes from one of the first reports: more than twenty years ago the Kornblihtt group showed that alternative exon inclusion is greatly affected by promoter identity. To demonstrate this, mammalian cells were transfected with plasmids containing a given RNAPII promoter and an alternative exon surrounded by constitutive exons, a so called AS reporter minigene. The only difference between these minigenes was the promoter, each of them giving place to identical pre-RNA molecules. After total RNA purification, retro-transcription and PCR amplification of AS isoforms, it was observed that the ratio of exon inclusion/skipping was drastically affected by promoter identity, demonstrating an intimate connection between transcription and pre-RNA processing (Cramer *et al.*, 1997). Moreover, transcription by an RNAPII lacking its carboxy-terminal domain (CTD), a repetitive structure rich in amino acids subject to post-translational modifications (Corden *et al.*, 1985), also affects AS regulation (de la Mata and Kornblihtt, 2006; Muñoz *et al.*, 2010). In fact, mutations in the CTD sequence that prevent or mimic its phosphorylation, by replacing serines with alanines or glutamic acids respectively, also modulate AS patterns (Muñoz *et al.*, 2009). These results demonstrate that, although the catalytic activity of RNAPII is not located in the CTD, the phosphorylation state of this domain is crucial for pre-RNA processing. Moreover, the role of transcription in the control of AS was further confirmed by the finding that transcription factors (Kadener *et al.*, 2001; Nogues *et al.*, 2002), coactivators (Auboeuf *et al.*, 2004), transcription enhancers (Kadener *et al.*, 2002), as well as chromatin remodelers (Batsche *et al.*, 2006) and factors that alter chromatin structure (Schor *et al.*, 2009; Luco *et al.*, 2010; Saint-Andre *et al.*, 2011), modulate AS. Coupling of transcription and AS most likely occurs through different factors interacting with a particular state of post-translational modification of the CTD. Then, the transcriptional complex can, in turn, either directly affect splice site selection or the speed (i.e., elongation rate) of the polymerase. According to the kinetic coupling model, a slow elongation rate of the transcribing RNAPII favors co-transcriptional recognition of a weak splice site, or other significant sequence in the pre-mRNA, before a stronger site located downstream is synthesized (Kornblihtt *et al.*, 2013). We have shown that UV exposure induces the hyperphosphorylation of the CTD, which reduces RNAPII elongation rates, thus affecting splice site selection of several genes, some of which are key for survival/apoptosis decisions (Muñoz *et al.*, 2009).

### Regulation of alternative splicing in a genotoxic scenario

Exposure to regular DNA damaging agents such as UV radiation, chemicals, or oxidative stress may affect every human gene. As an example, treating cells in culture with 20 J/m^2^ of UV light (254 nm) induces 4 photoproducts every 10 kbp, enough to damage every single one of the 21,000 human genes (van Hoffen *et al.*, 1995). In any case, upon exposure to UV radiation, some of these genes are less expressed, other genes are more expressed, and a bigger group remains unaffected (Muñoz *et al.*, 2017). Although it is clear that DNA damage *in cis* affects gene expression by at least altering the pace of a single transcribing RNAPII molecule in a damaged template (Geijer and Marteijn, 2018), *in trans* signaling does also take place and, therefore, we should pay attention to the different molecular mechanisms activated by DNA damage in order to understand how gene expression is regulated.

The fact that UV light affects AS *in trans* was demonstrated by at least two simple experiments. Firstly, transfection of pre-irradiated cells with an AS reporter minigene elicited similar AS patterns as those obtained when irradiating cells after transfection, demonstrating that UV light can control AS *in trans* (Muñoz *et al.*, 2009). Secondly, transfection of an *in vitro* UV-irradiated plasmid, with no transcriptional units or relevant sequences for mammalian cells, mimicked the AS patterns of UV-treated cells in AS reporter minigenes, therefore showing again, but by other means, that UV-induced DNA damage *in cis* is not necessary to induce the UV effect on AS (Muñoz *et al.*, 2017). Therefore, UV light, and in particular UV-induced DNA damage, affects gene expression *in trans.*

In the past few years we learned a simple lesson when studying gene expression control in a genotoxic scenario: there may be new functions for old players. While members of class-IV phosphoinositide 3-kinase (PI3K)-related kinase (PIKK) family, such as Ataxia-Telangiectasia-Mutated (ATM), Ataxia Telangiectasia and Rad3-related (ATR), and DNA-dependent Protein Kinase (DNA-PK), have well established roles in the response to different types of DNA damage (Awasthi *et al.*, 2015), novel roles in the control of gene expression under stress where recently described (Tresini *et al.*, 2015; Muñoz *et al.*, 2017; Liu *et al.*, 2019). Below, we will briefly discuss recent evidence showing the contribution of these DDR kinases to gene expression regulation.

The involvement of ATR in the control of AS upon UV irradiation was demonstrated by our group using human keratinocytes, the most abundant cell type in the skin. Nucleotide Excision Repair (NER) is one of the most versatile DNA repair systems in human cells, dealing with lesions induced by UV light, chemicals and some forms of oxidative damage (Nouspikel, 2009). We found that single stranded DNA (ssDNA) exposed during NER-dependent repair of UV-induced cyclobutane pyrimidine dimers (CPDs) activates the ATR kinase, which indirectly affects the phosphorylation state of RNAPII’s CTD (Muñoz *et al.*, 2017). The UV effect is enhanced by inhibition of gap-filling DNA synthesis, the last step in NER, supporting the notion of a role for ssDNA in the activation of an ATR-dependent signaling cascade controlling CTD phosphorylation and gene expression. Global Genome NER (GG-NER) is the branch of NER active throughout the whole genome and not just in transcriptionally active genes, as is the case for Transcription Coupled NER (TC-NER). As the UV effect on gene expression was reduced in the absence of DDB2/XPE, the main GG-NER sensor of CPDs, we proposed that less recognition would generate less ssDNA intermediates and consequently a decreased UV effect on gene expression (Muñoz *et al.*, 2017; Cambindo Botto *et al.*, 2018).

On the other hand, the role of ATM in the control of gene expression has been reported using human fibroblasts (Tresini *et al.*, 2015). They showed that a transcription-blocking DNA lesion induces spliceosome displacement from the nascent pre-RNA, therefore increasing R-loop formation which, in turn, activates ATM. According to the authors, activation of ATM further regulates spliceosome displacement, thus affecting gene expression globally (Tresini *et al.*, 2015).

Finally, a role for DNA-PK in the control of AS was recently suggested upon double strand break (DSB) induction. DSBs are repaired throughout the cell cycle by the non-homologous end joining (NHEJ) pathway, in which DNA-PK has a paramount role. The authors found that DNA-PK co-localizes with nuclear speckles, dynamic nuclear structures enriched in splicing factors, and its inactivation affected a set of AS events (Liu *et al.*, 2019).

### Alternative splicing regulation of DDR

As mentioned earlier, AS affects the expression of nearly the entire genome, and factors involved in every aspect of the DDR are not the exception. Members of the p53, Mdm, bcl-2, or caspase families of genes, among many others, are modulated by AS, affecting key aspects of the encoded products, such as their activity, localization, or half-life (Giono *et al.*, 2016). Consequently, DNA repair, the control of cell cycle, or the induction of cell death are key mechanisms regulated by AS and, not surprisingly, their misregulation can drive to cellular transformation (Shkreta and Chabot, 2015). The best-documented examples are Bcl-x (Muñoz *et al.*, 2009) and Caspase 9 (Shultz *et al.*, 2010), whose AS variants can promote or inhibit the apoptotic pathway. Although in the vast majority of cases the functional impact of the different AS isoforms is missing, evidence showing how AS regulates different aspects of the DDR accumulates. For instance, it has been shown recently that PRMT5, an arginine methyl transferase, controls AS patterns of the homologous recombination (HR) factor Tip60 altering the balance between HR and non-homologous end joining (NHEJ) (Hamard *et al.*, 2018), and that SIRT1, a NAD-dependent protein deacetylase, regulates AS patterns of different DDR factors (Wang *et al.*, 2018). Also, different splicing variants of BRCA1, a well know cancer susceptibility gene, modulate DNA repair mechanisms (Sevcik *et al.*, 2013), and the BRCA1 protein has been shown to be a regulator of the splicing process, affecting the expression of repair factors (Savage and Harkin, 2015). Moreover, as it has been documented in other reviews (Shkreta and Chabot, 2015), mRNA isoforms of the cell cycle CDC25B phosphatase (Baldin *et al.*, 1997) and the DDR kinase CHK2 (Berge *et al.*, 2010) display dominant-negative effects.

Finally, having in mind that transcription promotes not only opening of the DNA double helix and exposure of ssDNA, but also changes in DNA supercoiling, nucleosome occupancy, and collisions with replisomes in S phase (Bermejo *et al.*, 2011), it is not surprising that the transcriptional process by itself acts as a DNA damaging agent. The fact that highly expressed genes show high levels of mutagenesis is one of the many hints demonstrating that transcription affects the integrity of DNA (Kim *et al.*, 2007; Svejstrup, 2010). Therefore transcription, an essential process for life, also acts as a DNA damaging agent. While it is clear that transcription regulates alternative splicing, it is also clear that alternative splicing regulates transcription. In an elegant study, the Svejstrup laboratory recently showed that transcription, and therefore the genome’s damage load, is regulated by alternative splicing, since the ASCC3 gene generates isoforms that are able to favor (ASCC3 short isoform), or inhibit (long isoform) transcription recovery after DNA damage (Williamson *et al.*, 2017). Upon UV irradiation, general transcription is shut down (Rockx *et al.*, 2000), and RNAPII elongation rate decreases (Muñoz *et al.*, 2009), favoring the expression of shorter alternative splicing variants (Williamson *et al.*, 2017). After some time, during which DNA damage load partially decreases due to repair, transcriptional re-start is favored, since low elongation rates favor the expression of the ASCC3 short isoform. A possible interpretation is that in a DNA damage scenario global transcription should be shut down. This is not to prevent mRNA mutations, but to avoid transcription-associated damage in an already damaged DNA.

## Concluding remarks

**Figure 1:**
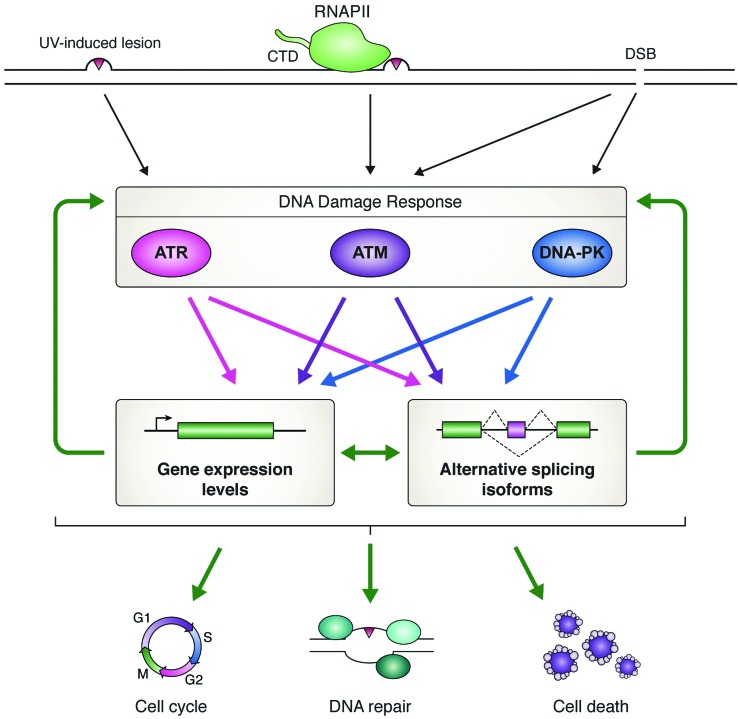
Roles recently reported for the kinases ATR, ATM and DNA-PK are depicted. UV-induced DNA lesions activate ATR and ATM, the latter being activated in particular by stalling of transcribing RNAPII. Double strand breaks (DSB) activate ATM and DNA-PK. These three central DDR kinases modulate gene expression globally, both by regulating gene expression levels and by modifying AS patterns. This modulation, in turn, tunes the DDR by modulating cell-cycle regulation, DNA repair and cell death.

Since AS is modulated by DNA damage and, in turn, modulates the response to a genotoxic agent (Figure 1), it is of interest to specifically manipulate AS patterns of key genes that may offer a benefit from a clinical point of view. As with the successful therapies using modified oligonucleotides to prevent the usage of a splice site in the treatment of spinal muscular atrophy (Wang *et al.*, 2018), the challenge is to identify specific AS isoforms whose expression may help to prevent cellular transformation or enhance cell death.
